# Spatiotemporal brain dynamics in 8-to-9-year-old children: A comparative study between preterm and term schoolchildren

**DOI:** 10.1016/j.nicl.2026.103949

**Published:** 2026-01-16

**Authors:** Solange Denervaud, Paola Zanchi, Céline J Fischer Fumeaux, Cléo Huguenin-Virchaux, Laureline Besuchet, Patric Hagmann, Anita C Truttmann, Juliane Schneider

**Affiliations:** a Clinic of Neonatology, Department of Mother Woman Child, CHUV, University of Lausanne, Lausanne, Switzerland; b CIBM Center for Biomedical Imaging, Switzerland; c MRI imaging and technology, Polytechnical School of Lausanne, Swiss Federal Institute of Technology Lausanne (EPFL), 1015 Lausanne, Switzerland; d The Sense Innovation and Research Center, Lausanne and Sion, Switzerland; e Clinic of Neuroradiology, Department of Radiology, CHUV, University of Lausanne, Lausanne, Switzerland

## Abstract

Preterm birth disrupts critical phases of brain maturation, placing individuals at increased risk for long-term cognitive and functional impairments. This study investigated how very preterm birth affects the spatial and temporal organization of functional brain networks in school-aged children born very preterm using a spatiotemporal connectome framework. Multimodal MRI, including diffusion-weighted imaging and resting-state fMRI, was acquired from 25 children born before 30 gestational weeks and 25 age- and sex-matched full-term controls (8–9 years). We characterized the structure–function coupling of dynamic brain activity through Connected Components (CCs) defined as structurally constrained sets of functionally co-active regions identified on a multilayer graph. Three different metrics were computed: CC number (count of distinct co-activation patterns), CC height (peak number of regions within a CC, representing the spatial extent) and CC width (temporal span across consecutive time repetitions (TRs)). In addition, we quantified System Diversity (SD) and Spatiotemporal Diversity (STD), indices reflecting integrative richness and temporal variability of functional network dynamics. CC number decreased with age across groups, reflecting typical developmental patterns, while CC height was significantly greater in preterm children and positively associated with processing speed, suggesting altered or compensatory network co-activation. No significant group differences were observed for SD metrics. However, network-level analyses revealed significantly lower STD values in all functional networks in the preterm group, indicating possible heightened temporal stability and reduced functional flexibility. These findings suggest that very preterm birth selectively alters the dynamic engagement of functional systems, with potential implications for cognitive vulnerabilities. (243 mots; 250 max).

## Highlights

• Spatiotemporal connectomics reveals altered brain dynamics after very preterm birth.
• Preterm children show increased spatial co-activation of functional networks.
• Temporal stability is elevated across all functional systems in preterm children.
• Global integrative capacity is preserved despite altered network dynamics.
• Altered temporal dynamics may underlie cognitive vulnerability after prematurity.

## 1 Introduction

Preterm birth, defined as delivery before 37 weeks of gestation (Blencowe et al., 2013) and affected approximately 9.9 % of live births in 2020. Of these, very preterm neonates (i.e., born before 32 weeks gestation) who constitute 15 % of all preterm births (Ohuma et al., 2023), face the highest risk of adverse outcomes (Volpe & Inder, 2024). Despite significant improvements in neonatal care, very preterm children show a consistently high prevalence of lifelong cognitive and motor impairments. For instance, moderate to severe cognitive or motor delay affects around 10.9 % and 12.0 % of infants born at 26–27 weeks, and 5.8 % and 6.3 % of those born at 28–32 weeks, respectively (Song, 2022). Longitudinal studies also confirm that preterm children (aged < 18 years) consistently exhibit lower IQ scores compared to term-born peers, a disparity remaining stable over the past four decades (Behboodi et al., 2025).

This persistent vulnerability is attributed to the abrupt interruption of critical brain maturation processes during the third trimester, a period marked by rapid synaptogenesis, dendritic arborization, myelination, and the emergence of large-scale neural networks (Bystron et al., 2008, Volpe and Inder, 2024). Exposure to the extrauterine environment during this highly sensitive window results in heightened cerebral vulnerability (Bouyssi-Kobar et al., 2016, Inder et al., 2023).

While the incidence of severe prematurity-related brain injuries, such as intraventricular hemorrhage and cystic periventricular leukomalacia (Inder et al., 2023), has markedly decreased, subtle microstructural and functional abnormalities are now more commonly recognized in preterm infants. These include delayed cortical maturation, impaired development of long-range white matter tracts, and disruptions in subcortical and associative network connectivity. For example, abnormalities in cerebellar structure and white-matter microstructure near term age are predictive of poorer cognitive and motor scores at 18–22 months of age (Rose et al., 2015). By early school age (6 years old), preterm children demonstrate atypical motor activation patterns and reduced large-scale resting-state network coherence (Urriola et al., 2025), along with reduced white-matter integrity, especially in the anterior thalamic radiation, linked with impairments in attention and processing speed performance between 9–11 years old (Li et al., 2025).

Crucially, neurodevelopmental outcomes following preterm birth are heterogeneous, reflecting a spectrum of brain maturation trajectories shaped by gestational age, medical complications, and environmental factors (Thalhammer et al., 2025, Nivins et al., 2025, Dimitrova et al., 2020). Advanced neuroimaging techniques, particularly diffusion-weighted imaging (DWI) and resting-state functional MRI (rs-fMRI), have been instrumental in identifying long-lasting alterations in both structural and functional connectivity in preterm-born infants relative to full-term controls. At term-equivalent age, preterm infants showed reduced thalamic volume and widespread white-matter abnormalities, with particular vulnerabilities in thalamocortical and subcortical-cortical pathways. Disruption in these pathways predict later motor, cognitive, and socioemotional difficulties (Ball et al., 2012, Brenner et al., 2021).

These atypical connectivity profiles often persist into later developmental stages and are associated with enduring cognitive and behavioral sequelae, such as attentional deficits and socio-emotional difficulties throughout childhood (Rogers et al., 2018). For example, semantic network analyses of verbal comprehension from the same cohort of very preterm children aged 8–10 revealed longer semantic distances, reduced network connectivity, and greater modularity, indicative of subtle disruptions in memory organization and lexical access (Décaillet et al., 2025).

Recent research underscores the dynamic and evolving nature of these effects, showing that preterm-related neurodevelopmental alterations change across childhood (Jansen et al., 2021) and adolescence, often paralleling shifting cognitive and behavioral outcomes (Kelly et al., 2022). These findings highlight the need for temporally sensitive analytical methods that can capture the evolving impact of prematurity on brain dynamics.

Furthermore, neural development throughout childhood is shaped by a complex interplay of intrinsic mechanisms and extrinsic influences, particularly sensory inputs, cognitive stimulation, and experience-dependent activities such as intense motor skill training (Ismail et al., 2017). These factors interact with intrinsic processes to shape the refinement of large-scale brain networks that underlie the emergence of high-level cognitive functions, such as attention control, working memory, and behavioral flexibility (Thomas et al., 2023). Despite substantial progress, much of the existing literature on preterm brain development relies on static measures of connectivity, which offer only limited insight into the dynamic nature of neural systems.

Conventional rs-fMRI studies typically assess functional connectivity as a temporally averaged measure, overlooking the brain’s intrinsic temporal variability. However, functional brain networks are inherently dynamic, continuously reconfiguring to support cognitive flexibility and adaptive behavior. A growing body of research has shifted toward dynamic functional connectivity analysis, which captures moment-to-moment fluctuations in interregional communication. One core principle in this field is metastability, defined as the brain’s capacity to alternate between integrated and segregated network configurations over time and believed to be fundamental for efficient cognitive processing (Deco et al., 2017).

To investigate these transient dynamics, emerging neuroimaging frameworks now integrate functional and structural data to model spatiotemporal connectomes. These approaches leverage diffusion-based tractography and time-resolved rs-fMRI to characterize how anatomical constraints shape dynamic patterns of functional co-activation. Using structural connectivity derived from diffusion-weighted imaging in combination with functional data allows to further increase the specificity of the brain network dynamics. By limiting the transient co-activation patterns to anatomically realistic connections, this approach reduces the risk of unreliable functional associations and highlights physiologically meaningful interactions. This method is particularly relevant for studies of very preterm children, whose long-range white matter tracts are especially vulnerable and mature more slowly than in full-term peers (Inder et al., 2023). Within this framework, two complementary metrics have been introduced: System Diversity (SD) and Spatiotemporal Diversity (STD). SD quantifies, via Shannon entropy, the variety of canonical functional systems (Yeo-7) that co-occur within Connected Components (CCs; structurally constrained sets of functionally co-active regions identified on a multilayer graph) over time. Therefore, higher SD reflects richer integrative repertoire (the engagement of a wider variety of distinct functional systems). In contrast, STD measures the temporal duration of each CC spatial pattern, reflecting how flexibly (high STD) or persistently (low STD) brain regions are recruited into functional assemblies (Griffa et al., 2017). This framework was applied in healthy adults (mean age 28.8) (Griffa et al., 2017), and later used to gain a developmental perspective on brain dynamic development (Vohryzek et al., 2020). In that context, it was unveiled that maturation is characterized by increasing SD, indicating greater inter-network integration, and decreasing STD, reflecting improved stability and efficiency of functional coordination (Vohryzek et al., 2020). These changes support the emergence of cognitive control and are thought to underlie improvements in executive function, attentional regulation, and information processing in school students aged between 4–18 years old (Zanchi et al., 2024). Given the known vulnerability of these systems in children born very premature (Niutanen et al., 2022, Taylor and Clark, 2016), the application of spatiotemporal connectome analysis may provide critical insight into the enduring neurobiological effects of early adversity. Notably, sensorimotor systems mature earlier in development (Hoff et al., 2013), whereas higher-order associative networks, including the frontoparietal and the salience systems, undergo prolonged postnatal maturation and especially involved in behavioral self-regulation (Gao et al., 2015). These later-developing systems may be particularly susceptible to perturbation in the context of preterm birth (Wehrle et al., 2018).

In this study, we employed a spatiotemporal connectome framework to investigate differences in brain network dynamics between children born very prematurely and their full-term peers at 8–9 years of age. Although dynamic functional connectivity has been characterized in preterm neonates (França et al., 2024) and multimodal approaches combining task-based and resting-state fMRI have been applied in school-age preterm children (Urriola et al., 2025), few studies have simultaneously applied dynamic, multimodal approaches to examine functional reorganization in preterm children at school age. Using a multimodal MRI approach that integrates DWI and time-resolved rs-fMRI, we aimed to elucidate the factors shaping spatiotemporal brain organization. Specifically, we conducted statistical analyses to examine whether concomitant structural and functional neural signals, referred to as Connected Components (CC) measures, including the number, height, and width of CC, were influenced by birth status (preterm vs. full-term), age, and sex. As an exploratory analysis, we also investigated relations between CC measures and cognitive outcomes in the preterm group, focusing on executive function, processing speed, verbal comprehension, and motor abilities, using correlational statistics.

In parallel, we quantified SD and STD, which are, as stated earlier, two complementary indices that capture, respectively, the richness and temporal variability of functional brain organization. Based on previous reports of reduced cognitive flexibility and attention-related difficulties in children born preterm (Taylor & Clark, 2016), we hypothesized that the preterm group would exhibit either lower SD, indicative of less differentiated network recruitment, or altered STD, reflected in overly stable patterns of functional activation (i.e., hyper-engagement of specific networks). We expected these deviations to be most pronounced in visual (VIS), somatomotor (SM), ventral attention (VA), dorsal attention (DA) and frontoparietal (FP) networks, systems that are either early-developing or highly sensitive to postnatal experiences, and which support key cognitive and motor functions often disrupted in children born very preterm. These alterations may reflect persistent neurodevelopmental disruptions and offer mechanistic insight into the cognitive and behavioral challenges commonly observed in this high-risk population.

## 2 Methods

### 2.1 Participants

All procedures were conducted in accordance with the Declaration of Helsinki and were approved by the Ethics Committee of the University Hospital of Lausanne (CER-VD, Switzerland). Preterm participants (born < 30 weeks gestation) were part of a cohort study recruited at birth between February 2011 and May 2013. During their routine neurodevelopmental follow-up visit at the Lausanne University Hospital (CHUV), they were invited to take part in the current study at 8–9 years of age. Data from a group of full-term born children (defined as born at ≥ 37 weeks of gestation, as reported by parents) from a parallel study run at CHUV were used. They were selected to be matched for age, sex, and socio-demographic background, as a reference group (Table 1).

**Table 1:** Demographic characteristics of preterm and full-term children.

**Control variable**	**Preterm(n = 25)**	**Term(n = 25)**	**Statistics**
Age (mean, SD)	8.83 (0.51)	8.75 (0.80)	U = 271, *p* = 0.42
Age (min–max)	8.0–10.0	7.30–10.5	
Sex ratio (f/m)	13 / 12	13 / 12	Chi^2^ = 0.00, *p* = 1.00
Socioeconomic status (SD)	3.17 (1.19) ^(2)^	3.10 (0.55) ^(1)^	NA
Fluid intelligence (SD)	107.95 (12.08) ^(1)^	32.36 (3.2)	NA
FD (SD)	0.297 (0.220)	0.535 (0.541)	U = 227, p = 0.097
FD (min–max)	0.107–0.780	0.099–2.336	

SD stands for standard deviation; f/m stands for female/male; FD stands for framewise displacement. (1): missing value for one participant; (2): missing values for two participants.

Written informed consent was obtained from the parents or legal guardians of all participants, and verbal or written assent was obtained from the children. Each participant received a voucher (approximately USD 40) as compensation. Data of 50 participants (mean age ± SD = 8.83 ± 0.51 years) were available for the analysis.

### 2.2 Behavioral data acquisition

In the preterm group, parental socioeconomic status (SES) was assessed using the Largo score, based on maternal education degree and paternal employment status, which ranges each from 1 to 6, 1 representing the higher level and 6 the lowest (Largo et al., 1989). Neuropsychological testing was performed using several standardised tests. Fluid intelligence (FI) was measured using the Fluid Reasoning Index (FRI) item of the Wechsler Intelligence Scale for Children (WISC-V) battery (Wechsler, 2016), a non-verbal assessment widely used to estimate general cognitive functioning. The test consists of two primary subtests: *Matrix Reasoning*, where the participants select the missing piece to complete matrix patterns that increase in difficulty, and *Figure Weights*, where the participants balance scales by choosing the correct item from a list and placing it on the empty side. The items become more challenging as the test progresses. The combined score from these two subtests is then converted into a standard score. Two other indices from the WISC were collected; the Processing Speed Index (*PSI*) and the Verbal Comprehension Index (*VCI*). Fine motor coordination was assessed using the corresponding subtest of the Beery Developmental Test of Visual-Motor Integration (Beery & Beery, 2010). Executive functioning was evaluated with a parental questionnaire, the Behavior Rating Inventory of Executive Function (BRIEF, Gioia et al., 2013), using the Global Executive Composite score. Furthermore, clinical variables were collected as part of the routine follow-up examination, including perinatal characteristics (birth gestational age and weight, Apgar score reflecting neonatal adaptation, CRIB score indicating early mortality risk within the first 24 h after admission) and demographics at follow-up at 8–9 years of age (i.e., parental socioeconomic status as measured by the Largo score), see Table 2.

**Table 2:** Descriptive variables of the preterm group.

**Variable**	**Mean (SD)**	**Min − Max**	**Shapiro-Wilk test**
Gestational age (week)	27.5 (1.40)	25.1–31.6	*W* = 0.942, *p* = 0.168
Birth weight (g)	897 (227)	610–1590	*W* = 0.891, *p* = 0.012
Apgar 5 min	7.00 (1.50)	3–9	*W* = 0.904, *p* = 0.022
CRIB score	3.71 (2.65) ^(1)^	1–10 ^(1)^	*W* = 0.882, *p* = 0.009 ^(1)^

(1): missing value for one participant.

In the full-term reference group, parental SES was assessed using a validated paper-based questionnaire that included items on employment status, occupational classification, and educational attainment, with higher composite scores indicating higher SES (Genoud, 2011). FI was evaluated using the black-and-white version of Raven’s Progressive Matrices (PM-47; Raven & Raven, 2003), a standardized non-verbal reasoning test. The task comprises 36 matrix items that progressively increase in complexity and targets two core components of intelligence: reproductive ability, defined as the capacity to retain and retrieve information, and deductive reasoning, reflecting the ability to process and interpret complex patterns. Participants were given 15 min to complete the test, and total scores were used as an index of FI, with higher scores indicating greater fluid intelligence.

### 2.3 MRI data acquisition

Neuroimaging data were acquired at the Lemanic Biomedical Imaging Center (CIBM) of the University Hospital Lausanne, using a Siemens 3 T Prisma-Fit scanner equipped with a 64-channel head coil. Participants’ heads were stabilized using foam padding to minimize motion artifacts. The first author was present and helped ensure homogeneity of acquisitions.

The scanning protocol consisted of three imaging modalities. Structural data were acquired using a high-resolution three-dimensional T1-weighted MPRAGE sequence (time repetition (TR) = 500 ms, TE = 2.47 ms, flip angle = 8°, voxel size = 1 mm^3^, 208 slices). Diffusion data were obtained using a Diffusion Spectrum Imaging (DSI) sequence, divided into four recordings, with 1.6 mm isotropic resolution, 129 diffusion-encoding directions, a b-value maximum of 3000 s/mm^2^, and whole-brain coverage over approximately 13 min. Functional data were acquired using a resting-state BOLD fMRI sequence (open eyes) with gradient echo-planar imaging (voxel size = 2.2 × 2.2 × 3 mm^3^, TR = 500 ms, TE = 33 ms, 720 volumes), with a total acquisition time of 6 min.

### 2.4 MRI preprocessing

First, regarding the structural MRI data, T1-weighted images were preprocessed using FreeSurfer v5.1.0 (http://surfer.nmr.mgh.harvard.edu/) for cortical and subcortical segmentation. The brain was parcellated into 504 regions of interest (ROIs) based on the Lausanne 2018 symmetric atlas (included in Connectome Mapper 3; Tourbier et al., 2020) Second, diffusion MRI data were processed using the Connectome Mapper 3 pipeline, which includes motion correction, tensor reconstruction, and deterministic whole-brain streamline tractography. Structural connectivity matrices were generated by counting the number of streamlines between each pair of ROIs. A group-level structural template was created by binarizing individual matrices and retaining only connections present in at least 50 % of participants.

Finally, functional data were preprocessed using fMRIPrep (Esteban et al., 2019), including slice realignment, head motion correction, spatial normalization to MNI space (ICBM 152 Nonlinear Asymmetric template 2009c), and co-registration to anatomical space. Independent Component Analysis (ICA)-based Automatic Removal of Motion Artifacts (ICA-AROMA; (Pruim et al., 2015) was used to reduce noise. Slice-timing correction was omitted due to the use of multiband acquisition.

Functional time series were extracted for each ROI by averaging the BOLD signal within each anatomical region, after mapping the Lausanne 2018 atlas to the Montreal Neurological Institute (MNI) space. The entire preprocessing pipeline was performed by the first author to ensure consistency in the application of the software pipelines.

### 2.5 Spatiotemporal connectome Construction

To estimate the spatiotemporal connectome, we combined functional time series with the group level structural connectivity template following the method described by Griffa et al. (Griffa et al., 2017). Each time frame was defined as one TR (500 ms), corresponding to a single time point in the BOLD fMRI signal. Functional time series were z-scored and binarized using a threshold of ± 2 standard deviations (Tagliazucchi et al., 2012).

A multilayer graph was then constructed in which each layer represented the brain network at a single time frame. Within-layer edges were created between two nodes (i.e., brain regions) only when two conditions were met simultaneously: (i) the regions were structurally connected according to the structural template, and (ii) both regions displayed supra-threshold BOLD activity (±2 SD) at the corresponding time point. To model temporal dynamics, between-layer edges were added by connecting each region to itself in the subsequent time frame, ensuring temporal continuity and enabling activity to propagate across the layers.

From the resulting multilayer graph, Connected Components (CC), defined as sets of nodes that are both structurally connected and functionally co-active over time, were then identified by applying a standard graph-theoretic connected-component decomposition on the multilayer graph; no clustering was used. For each participant, the total number of CCs (total count of distinct CCs) was measured, and for each CC, height (peak number of node count within a CC, reflecting the spatial extent) and width (number of consecutive layers spanned, reflecting temporal duration) were quantified.

The entire processing pipeline for the CC extraction was performed by the second first author to ensure consistency in the application of the software pipelines.

### 2.6 Computation of System and spatiotemporal Diversities

We quantified the dynamic reorganization of brain networks using two complementary metrics as defined in the spatiotemporal connectome framework (Vohryzek et al., 2020). First, the System Diversity (SD) coefficient, which measures the heterogeneity of recruitment across functional systems, was computed. Each node in a CC was labeled based on its assignment to one of the seven canonical Yeo networks: visual (VIS), somatomotor (SM), dorsal attention (DA), ventral attention (VA), limbic (LIM), frontoparietal (FP), and default mode (DM) (Yeo et al., 2011). For each CC, the probability distribution over these systems was computed, and SD was calculated as the Shannon entropy of this distribution. Second, the spatiotemporal Diversity (STD) coefficient was calculated to quantify the temporal variability of functional engagement. Each CC was represented as a normalized spatial activation vector (length = number of ROIs), with weights corresponding to the frequency of activation. The STD was computed as the average cosine similarity between all pairs of CC, providing a measure of how consistently the same spatial patterns recur over time.

SD and STD were computed for each functional system by including only CC in which ≥ 20 % of the nodes belonged to that system.

The entire processing pipeline for the extraction of SD and STD coefficients was performed by the second first author to ensure consistency in the application of the software pipelines.

### 2.7 Statistical analysis

#### 2.7.1 Behavioral measures

To compare children born prematurely to the full-term reference ones’ homogeneity in terms of age and sex ratio, a Student *t*-test and a Chi-square test were respectively computed. Given that both fluid intelligence levels were not measured using the same task (WISC-V Fluid Reasoning Index in the preterm group; Raven PM-47 in the full-term group), no between-group statistical comparison was performed. Descriptive statistics are reported separately for each test. Therefore, we cannot exclude that differences in fluid intelligence exist and may still influence the results. Normality of descriptive variables of the preterm group (Gestational age, Birth weight, Apgar 5 min and CRIB score) was assessed using the Shapiro-Wilk test.

Parental socioeconomic status (SES) was also assessed differently across groups. In the preterm group, SES was computed using the Largo score (Largo et al., 1989) by averaging the maternal and paternal score for each participant. In the reference group, a composite SES score was derived from a questionnaire (Genoud, 2011). As the two SES measures were not directly comparable, SES scores were converted into within-group percentile ranks separately for each group. For the preterm group, percentile values were inverted to account for the scoring direction (lower values indicating higher SES), whereas in the reference group higher values reflected higher SES. This procedure ensured that higher percentile values consistently represented higher socioeconomic status across groups. No statistical test was conducted between groups. Descriptive statistics are reported separately, see Table 1.

For the other cognitive and behavioral outcomes: executive function (BRIEF-GEC), processing speed (WISC-PSI), verbal comprehension (WISC-VCI), and fine motor coordination (Beery-MC), scores were analyzed within the preterm group only. These scores were used in exploratory Pearson correlations with brain connectivity features.

#### 2.7.2 Connected Components

First, statistical analysis has been conducted to examine whether brain connectivity measures (number, height, and width) of the CC were influenced by the participants’ birth (premature vs. full-term), age and sex. Accordingly, three analyses of covariance (ANCOVAs) were conducted for each connectivity metric of the connected components (CC): number, height, and width. The CC metrics served as the dependent variable, and each model included the birth status (premature vs. full-term) as a fixed factor, sex as an additional fixed factor, and age and SES as a covariate. As an additional check, framewise displacement (FD) was also included as a covariate in the analysis. Interaction terms between group and age, between group and sex, between group and SES and between group and FD, were also included in the models to assess their potential effects.

Second, exploratory Pearson correlations were computed for the preterm group to investigate relation between CC features (i.e., number, height, width) and birth conditions (gestational age, weight, Apgar 5 min, CRIB), as well as between CC and cognitive outcomes, focusing on executive function (BRIEF-Global Executive Composite), processing speed (*WISC-PSI*), verbal comprehension (*WISC-VCI*), and fine motor coordination (Beery MC). These analyses are considered exploratory, and Benjamin-Hochberg FDR correction was applied alongside reporting two-sided *p*-values to control for multiple comparisons.

#### 2.7.3 Network-level dynamics (SD and STD)

To further explore the effects of preterm birth on brain dynamics, SD and STD values were computed at the global scale (whole brain) and the level of 7 resting-state functional networks and compared to those of full-term reference group. *P* values were calculated using permutation-based statistical testing against null distributions generated from 1,000 iterations (two-tailed permutation test). Bonferroni method was used to correct for multiple comparisons for the functional system analysis, which represents the primary family of tests. *P*-values were considered significant if *p* < 0.05.

## 3 Results

### 3.1 Behavioral variables

Preterm and full-term children were comparable in terms of age and sex ratio (all *ps* > 0.42). The results of the Shapiro-Wilk test highlighted that the FD measures were not normally distributed (Preterm group: W = 0.744, *p* < 0.001; Control group: W = 0.750, *p* < 0.001). Therefore, a Mann-Whitney *U* test was used to compare the two groups, indicating no significant difference (U = 227, p = 0.097) (see Table 1).

Concerning the descriptive variables of the preterm group, the results of the Shapiro-Wilk test indicated that the distribution of Gestational Age was consistent with normality (*W* = 0.942, *p* = 0.168). However, Birth Weight, Apgar 5 min score and CRIB score all exhibited statistically non-normal distributions (all *p* < 0.05) (see Table 2).

Preterm and full-term children were matched for age and sex, and while fluid intelligence and socioeconomic status were not comparable across groups, several neonatal variables in the preterm group showed non-normal distribution, reflecting expected clinical heterogeneity.

### 3.2 Connected Component

**Fig. 1:**
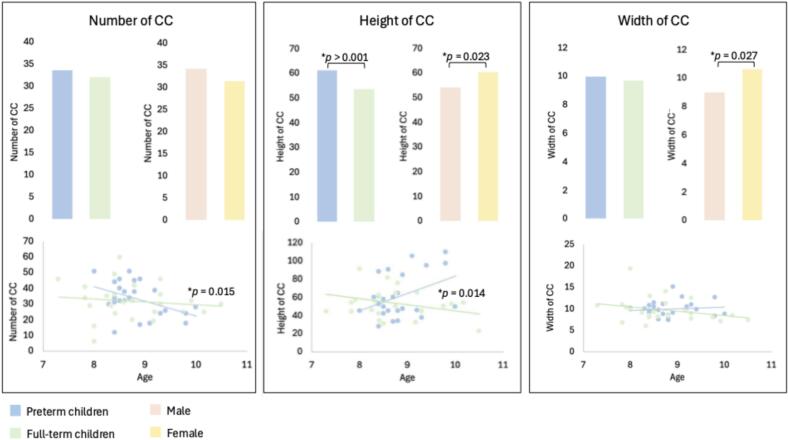
Number, height, and width of CC as a function of birth group (preterm vs. full-term) and sex (male vs. female), and their relation to age. Bar graphs show group means values for each condition and scatterplots show age-CC relationships Scatterplots illustrate the relationship between age and CC parameters, across groups. Number of CC: No significant main effects of group or sex were found. However, age significantly influenced the number of CC (*p* = 0.015), suggesting a developmental effect with decreasing CC number across age. Height of CC: A significant main effect of the birth group was observed (*p* < 0.001), with preterm children showing greater CC heights than full-term children, and a significant main effect of sex was discovered (*p* = 0.023), with females showing greater CC height compared to males. A significant interaction between group and age was also found (*p* = 0.004), indicating that the influence of age on CC height differs between groups. Width of CC: A significant main effect of sex was found (*p* = 0.027), with females showing greater CC width than males.

The results of the ANCOVA revealed that the number of CC was significantly influenced by age (*F*(1, 37) = 6.515, *p* = 0.015, η^2^ = 0.150), indicating a developmental effect. No significant main effect of group, sex, SES or FD or any interaction effects were found (all *ps* > 0.059), Figure 1, left panel.

The height of the CC was significantly influenced by the birth group (*F*(1,37) = 13.063, *p* < 0.001, η^2^ = 0.261), with children born prematurely showing greater CC heights compared to the full-term children. Significant main effects were also found for age (*F*(1, 37) = 6.642, *p* = 0.014, η^2^ = 0.152), and sex (*F*(1, 42) = 5.63, *p* = 0.023, η^2^ = 0.132). Additionally, a significant interaction was observed between group and age (*F*(1, 37) = 14.177, *p* < 0.001, η^2^ = 0.277), suggesting that the influence of age on CC height varied between groups. No other significant effects were identified (all *ps* > 0.124), Figure 1, central panel.

The width of the CC showed a significant main effect of sex (*F*(1, 27) = 5.322, *p* = 0.027, η^2^ = 0.126), with females presenting wider CC than males. No other significant effects or interactions were observed (all *ps* > 0.202), Figure 1, right panel.

Exploratory Pearson correlation analyses revealed a strong negative association between the number of CC and their height (r = -0.737, *p* < 0.001), as well as a significant negative correlation with the width of the CC (r = -0.414, *p* = 0.040).

Regarding the birth conditions, no significant correlations were observed between the birth variables and CC features (all *ps* > 0.387).

**Fig. 2:**
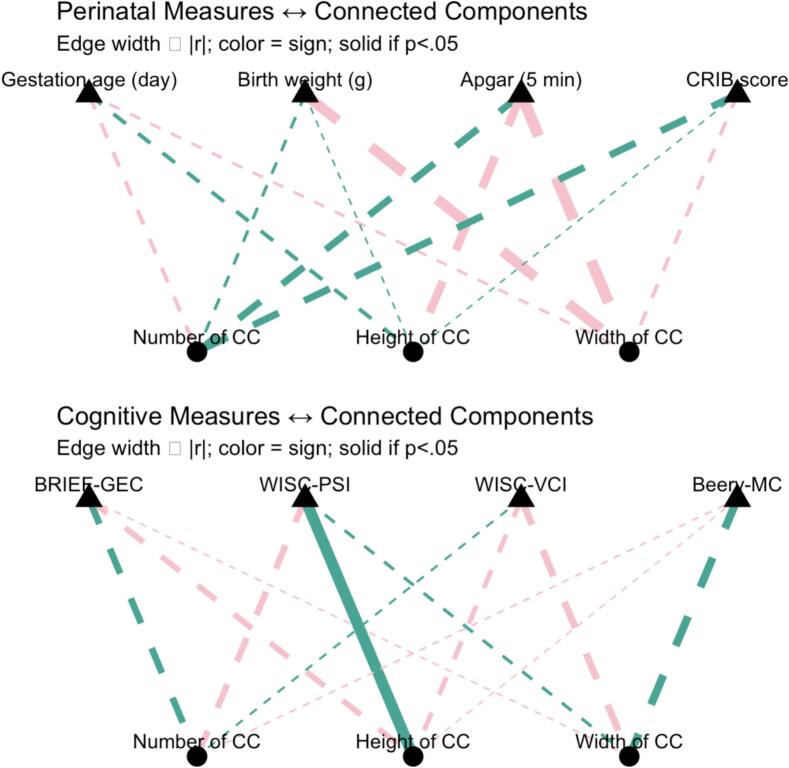
**Correlations between Connected Component (CC) characteristics and birth conditions and cognitive outcomes.** R-values are imaged as line width, positive and negative correlations are reported as pink and green colors respectively, and the significant relation is reported with a solid line. **Top panel:** Pearson correlation between number, height, and width of CC and birth conditions variables, including gestational age (in days), birth weight (in grams), 5-minute Apgar score and CRIB score. No significant correlations were found between CC measures and any birth conditions variable (all *ps* > 0.38). **Bottom panel:** Pearson correlation coefficients between CC characteristics and cognitive outcomes measures: BRIEF-GEC (executive function), WISC-PSI (processing speed), WISC-VCI (verbal comprehension), and Beery-MC (motor coordination). A significant positive correlation was observed between CC height and WISC-PSI scores (r = 0.418, *p* = 0.042), suggesting that increased CC height is associated with better processing speed performance. However, after adjusting for multiple comparisons using Benjamini-Hochberg FDR corrections, no correlation remained statistically significant (all *ps* > 0.924). No other significant correlations were found (all *ps* > 0.165). See Supplementary S1 and S2 for details. (For interpretation of the references to colour in this figure legend, the reader is referred to the web version of this article.)

In terms of cognitive outcomes, a significant positive correlation was found between the height of the CC and the processing speed index (WISC-PSI) (r = 0.418, *p* = 0.042), suggesting that greater height can be associated with improved cognitive processing speed. No other significant correlations were observed between CC metrics and cognitive scores (all *ps* > 0.130). After adjusting for multiple comparisons using Benjamini-Hochberg FDR corrections, no correlation remained statistically significant (all *ps* > 0.924), confirming the purely exploratory nature of these associations, Figure 2.

Connected Component metrics revealed a developmental decrease in component number with age, increased component height in preterm children, positively linked to processing speed while not robust to FDR correction, and sex-related differences in width, highlighting distinct spatial and temporal network characteristics across age, birth status, and sex.

### 3.3 Network-level dynamics (SD and STD)

To further investigate how preterm birth influences the spatial and temporal organization of brain activity, we examined the SD (integration) and STD (stability) across resting-state functional networks. By comparing prematurely born (P) and full-term reference (C) children against null distributions, the results revealed no significant difference at the whole-brain level for either STD (*p* = 0.292) or SD (*p* = 0.384).

**Fig. 3:**
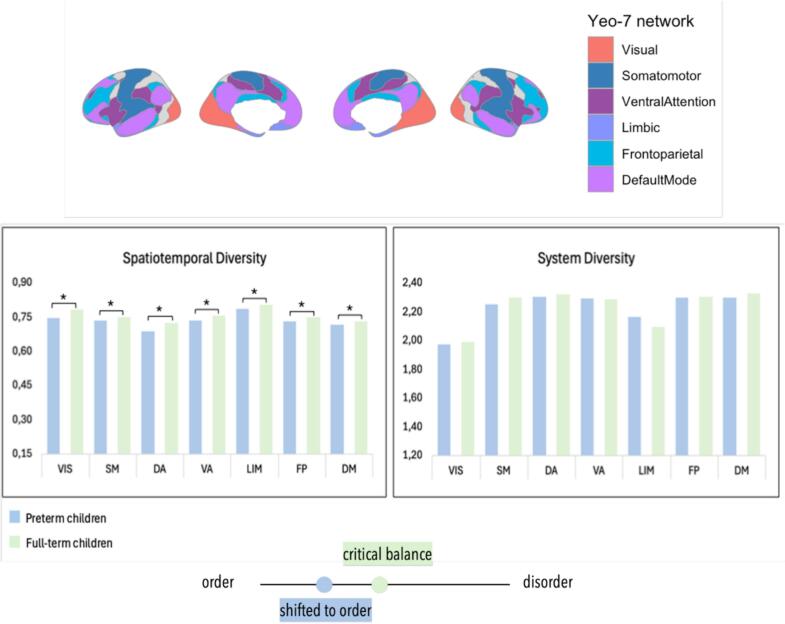
**Top panel:** Representation of the brain's main functional networks according to Yeo’s classification; visual (VIS), somatomotor (SM), dorsal attention (DA), ventral attention (VA), limbic (LIM), frontoparietal (FP), and default mode (DM) networks**. Middle panel:** Spatiotemporal (STD) and System Diversity (SD) across resting-state functional networks in preterm and full-term children. Left panel: STD reflecting spatiotemporal stability (STD) of brain activity. Significant group differences (*p* < 0.05 after Bonferroni-correction, indicated by *) were observed in the visual (VIS), somatomotor (SM), dorsal attention (DA), ventral attention (VA), limbic (LIM), frontoparietal (FP), and default mode (DM) networks, with preterm children showing greater temporal stability. Right panel: SD reflecting the integration nature of brain network dynamics, showed no significant differences between groups. **Bottom panel:** An abstract representation of meaning of excessive STD values suggesting a shift toward excessive order in brain network dynamics (i.e., possible inflexibility).

However, individual network analyses showed significant differences for STD measures. The visual (VIS, *p* < 0.0001), somatomotor (SM, *p* = 0.042), dorsal attention (DA, *p* < 0.0001), ventral attention (VA, *p* < 0.0001), limbic (LIM, *p* = 0.007), default mode (DM, *p* = 0.007) and frontoparietal (FP, *p* = 0.035) networks all showed significant difference between the two groups, indicating increased temporal stability in prematurely born children, see Figure 3, left panel.

Regarding the SD values, after the Bonferroni correction, all networks remained non-significant (all *ps* > 0.25), see Figure 3, right panel.

While no group differences were found at the whole-brain level, network-level analyses revealed significantly increased temporal stability (STD) across multiple functional systems in preterm children, suggesting altered dynamic regulation of brain activity despite preserved global integration (SD).

## 4 Discussion

This study examined how very preterm birth influences the spatial and temporal dynamics of functional brain networks in middle childhood using a spatiotemporal connectome framework. The preterm and full-term groups were matched for age and sex. However, fluid intelligence was measured using different instruments across groups, precluding direct statistical comparison, nor including this metric as covariate in our analyses. Therefore, we cannot exclude the fact that differences in overall fluid intelligence could have affected our results. Our findings suggest that children born very prematurely exhibit differences in the temporal stability, but not the integrative complexity of functional brain activity, within all large-scale networks.

Connected Component (CC) metrics provided insight into the evolving spatial and temporal architecture of dynamic brain organization. We found that the number of CC decreased significantly with age, consistent with studies showing that childhood and adolescence are marked by increased differentiation and modularity of brain networks, reflecting more refined and flexible network repertoires (Huang et al., 2015, Khundrakpam et al., 2013). Importantly, there were no group or sex differences in CC number, suggesting that the global expansion of functional network configurations with age is preserved in children born preterm.

In contrast, CC height was significantly greater in the preterm group, with a significant effect of age as well as a notable group-by-age interaction, indicating a distinct developmental trajectory in these children. This metric may reflect the degree of co-activation of functional configurations. Elevated CC height in preterm children may indicate intensified recruitment of functional assemblies, potentially pointing to reduced flexibility in disengaging active networks. This interpretation would be consistent with prior studies reporting less dynamic reconfiguration and greater temporal stability of functional connectivity in preterm and low-birth-weight populations (He and Parikh, 2015, Scheinost et al., 2016). Such alterations may reflect either compensatory adaptations in response to early disruptions or maladaptive rigidity in network function (Nosarti & Froudist-Walsh, 2016). More work is needed to unveil the cause and impact of increased CC height. So far, we further observed that within the preterm group, CC height was positively correlated with processing speed, even if not robust to FDR correction, suggesting that greater persistence of network states may support faster cognitive operations in some domains. This could reflect a compensatory enhancement in the efficiency of specific functional circuits that support basic information processing, which may be more plastic or resilient in preterm children. Notably, no associations were found between CC metrics and executive function, verbal comprehension, motor skills, or neonatal variables. This lack of broad correlation may stem from the heterogeneity in neurodevelopmental outcomes in preterm populations and underscores the need for larger samples to dissect individual differences more precisely (Luu et al., 2011, Montagna and Nosarti, 2016). A significant main effect of sex on CC height was also highlighted, with females showing greater CC heights compared to males, consistent with existing literature showing sex differences in functional connectivity patterns in children (Tomasi & Volkow, 2023).

Similarly, a significant sex effect on CC width was also observed, with females exhibiting broader CC than males. Although the interpretation of CC width remains exploratory, this finding aligns with evidence that female brains often show more diffuse and bilateral patterns of connectivity, potentially contributing to observed sex differences in cognitive strategies and developmental trajectories (Gur and Gur, 2016, Satterthwaite et al., 2014). However, further investigation is needed to determine the behavioral significance of this spatial difference in dynamic network configuration.

At the whole-brain level, we observed no significant group differences in System Diversity (SD) or Spatiotemporal Diversity (STD). These findings suggest that global integrative capacity and temporal variability of functional activity are largely preserved in preterm children by 8–9 years of age. However, region-specific analyses revealed significantly lower STD values in all networks in the preterm compared to the full-term group. This indicates a heightened temporal stability within the functional systems, which may reflect a globally reduced flexibility or excessive persistence of network activity. These findings align with recent evidence that preterm birth disrupts the dynamic balance of functional brain organization while preserving global integrative capacity (Fischi-Gomez et al., 2016; Padilla et al., 2020; Scheinost et al., 2016). For example, a recent study from Padilla et al. (2020) using intrinsic ignition and Hopf modeling demonstrated that extremely preterm children exhibit fewer ignition events, altered hierarchical propagation, and deviations from optimal criticality, particularly within rich-club hubs essential for large-scale integration. Similarly, our spatiotemporal connectome analyses revealed increased temporal stability and reduced flexibility of network states in preterm children, as reflected by elevated CC height and lower spatiotemporal diversity, despite preserved system diversity and age-related expansion of network repertoires.

Together, these results suggest that preterm birth does not primarily impair the structural–functional scaffolding necessary for integration but instead biases temporal dynamics toward greater persistence and reduced adaptability. Such rigidity may reflect compensatory adaptations that enhance efficiency in basic processing, as indicated by associations with processing speed, but at the cost of diminished flexibility in higher-order cognitive and motor domains. This interpretation is consistent with theoretical models emphasizing the role of metastability (i.e., oscillation between order and disorder) as a prerequisite for optimal information processing (Deco & Kringelbach, 2016; Tognoli & Kelso, 2014). Thus, by shifting the brain away from the critical point that normally enables rapid transitions between integrated and segregated states, preterm birth may constrain the dynamic range of functional activity, contributing to the attentional and executive vulnerabilities frequently observed in this population (Bogičević et al., 2021, Niutanen et al., 2022, Taylor and Clark, 2016). However, while our findings highlight group-level differences in spatiotemporal brain dynamics between preterm and term-born children, the limited sample size of the very preterm group prevented stratified analyses based on cognitive outcomes, which are likely to reflect meaningful within-group heterogeneity.

Several limitations should be acknowledged when interpreting the present findings. First, the sample size was modest, particularly for subgroup and correlational analyses, which may have limited statistical power to detect subtle effects or interactions, especially in the exploratory associations with cognitive performance. In addition, because socioeconomic status and fluid intelligence were assessed using different instruments across groups and could not be meaningfully harmonized, we were unable to include it as a covariate in our models, and therefore cannot rule out its potential confounding influence on the observed group differences. Larger cohorts with similar demographics and cognitive measures are needed to validate these findings and assess individual variability more robustly. Second, the cross-sectional design precludes conclusions about developmental trajectories; longitudinal studies are essential to determine whether the observed alterations in spatiotemporal dynamics persist, normalize, or evolve over time. Third, while the spatiotemporal connectome framework offers a powerful multimodal approach, its interpretation depends on several methodological choices that may influence the estimation of System and Spatiotemporal Diversity and warrant further standardization across studies. Finally, although groups were matched on age and sex, we did not control for other potential confounders such as educational factors (Zanchi et al., 2024), which may also shape neurodevelopmental outcomes. Future work incorporating these variables will be critical to disentangle biological from environmental contributions to altered brain dynamics in preterm populations.

In conclusion, by applying a multimodal, spatiotemporal connectome framework, this study advances our understanding of how preterm birth shapes the dynamic architecture of the developing brain. Importantly, it highlights the value of temporal network metrics, which may be more sensitive than static measures in capturing subtle, system-specific changes in brain organization. However, future research should focus on longitudinal trajectories to determine whether these dynamic alterations persist, resolve, or evolve during adolescence and how they relate to later functional outcomes. Larger samples and integration with genetic, environmental, and behavioral data may also help clarify the mechanisms underlying individual variability in resilience and risk among preterm populations.

## Data availability statement

Shared data include per-subject metrics of Connected Components (number, height, width), motion parameters, socioeconomic status, and cognitive summary scores available: https://gitlab.cibm.ch/denervaud/sdstd-neobrain.git.

## CRediT authorship contribution statement

**Solange Denervaud:** Writing – original draft, Visualization, Validation, Supervision, Resources, Investigation, Formal analysis, Data curation. **Paola Zanchi:** Writing – original draft, Visualization, Formal analysis. **Céline J Fischer Fumeaux:** Writing – review & editing, Project administration, Funding acquisition, Conceptualization. **Cléo Huguenin-Virchaux:** Writing – review & editing, Validation, Investigation, Data curation. **Laureline Besuchet:** Writing – review & editing, Validation, Data curation. **Patric Hagmann:** Writing – review & editing, Software, Methodology, Conceptualization. **Anita C Truttmann:** Writing – review & editing, Validation, Project administration, Conceptualization. **Juliane Schneider:** Writing – original draft, Validation, Supervision, Resources, Project administration, Investigation, Funding acquisition, Conceptualization.

## Declaration of competing interest

The authors declare that they have no known competing financial interests or personal relationships that could have appeared to influence the work reported in this paper.

## Data Availability

Data will be made available on request.
