# Supplementary material for: Spatiotemporal brain dynamics in 8-to-9-year-old children: A comparative study between preterm and term schoolchildren
Source: Neuroimage Clin. 2026 Jan 16;49:103949. doi: 10.1016/j.nicl.2026.103949 (PMC12859200; doi:10.1016/j.nicl.2026.103949)
Supplement: Supplementary Data 1 [file mmc1.docx]

**Supplementary**

**S1**

| Correlation Matrix | | | | |
| --- | --- | --- | --- | --- |
|  |  | Number CCs | Height CCs | Width CCs |
| Gestation age (day) | Pearson's r | -0.059 | 0.059 | -0.041 |
|  | df | 23 | 23 | 23 |
|  | *p*-value | 0.781 | 0.779 | 0.847 |
| Birth weight (g) | Pearson's r | 0.045 | 0.022 | -0.168 |
|  | df | 23 | 23 | 23 |
|  | *p*-value | 0.831 | 0.917 | 0.423 |
| Apgar (5 min) | Pearson's r | 0.109 | -0.134 | -0.181 |
|  | df | 23 | 23 | 23 |
|  | *p*-value | 0.602 | 0.523 | 0.387 |
| CRIB score | Pearson's r | 0.111 | 0.019 | -0.055 |
|  | df | 22 | 22 | 22 |
|  | *p*-value | 0.607 | 0.929 | 0.798 |

**S2**

| Correlation Matrix​ | | | | |
| --- | --- | --- | --- | --- |
| ​ | ​ | Number of CC​ | Height of CC​ | Width of CC​ |
| BRIEF-GEC​ | Pearson's r​ | 0.247​ | -0.211​ | -0.055​ |
|  | df​ | 17​ | 17​ | 17​ |
|  | *p*-value​ | 0.309​ | 0.385​ | 0.823​ |
| WISC-PSI​  ​ | Pearson's r​ | -0.230​ | **0.418​** | 0.112​ |
|  | df​ | 22​ | **22​** | 22​ |
|  | *p*-value​ | 0.279​ | **0.042​ *** | 0.603​ |
| WISC-VCI​ | Pearson's r​ | 0.077​ | -0.175​ | -0.256​ |
|  | df​ | 22​ | 22​ | 22​ |
|  | *p*-value​ | 0.721​ | 0.414​ | ​0.226 |
| Beery-MC​ | Pearson's r​ | -0.061​ | -0.050​ | 0.293​ |
|  | df​ | 22​ | 22​ | 22​ |
|  | *p*-value​ | 0.778​ | 0.817​ | 0.165​ |
